# Transosseous Fixation of the Distal Tibiofibular Syndesmosis: Comparison of Interosseous Suture and Endobutton Across Age Groups

**DOI:** 10.7759/cureus.40355

**Published:** 2023-06-13

**Authors:** Samantha Baxter, Eleanor Farris, Andrea H Johnson, Jane C Brennan, Elizabeth M Friedmann, Justin J Turcotte, David J Keblish

**Affiliations:** 1 Orthopedics, Anne Arundel Medical Center, Annapolis, USA; 2 Orthopedic Research, Anne Arundel Medical Center, Annapolis, USA; 3 Orthopedic Surgery, Anne Arundel Medical Center, Annapolis, USA

**Keywords:** complications, postoperative outcomes, syndesmotic repair, trimalleolar ankle fracture, suture bridge fixation

## Abstract

Background

In the ankle, suture bridge fixation for syndesmotic injuries is commonly employed. Initial recommendations for suture bridge constructs advised against using the device in patients with insufficient quantity or quality of bone. Therefore, many surgeons limit its use to younger, more athletic patients and use traditional screw fixation in older, less active patients. The purpose of this study is to compare the outcomes of suture bridge fixation for syndesmotic repair in patients ≥ 60 years old vs patients < 60 years old.

Methods

A retrospective review of 140 ankle fracture patients from a single institution who received suture bridge fixation between July 13, 2010, and February 2, 2022, was performed. Patient data was obtained from patient records in the electronic health record. Univariate analysis, including chi-square and independent t-tests, was used. Complications included delayed wound healing, infection, hardware loosening, and non-union.

Results

There were no significant differences in demographics, comorbidities, primary or other procedures, loss of fixation, and neuropathy between groups. There was also no difference within the distribution of the mechanism of injury, affected side, or Weber classification. Finally, the rate of complication and complication type showed no significant differences between patients 60 years and older versus 60 years and younger. Complication rates and types in patients > 60 years versus < 60 years were not significantly different.

Conclusion

The use of the suture bridge fixation in patients > 60 years may not lead to an increased risk of complications and appears to be safe for use.

## Introduction

The method for repair of ankle syndesmosis injuries is a highly studied topic and preferences for fixation differ among orthopedic surgeons. Injuries to the tibiofibular syndesmosis often occur from external rotation forces to the foot with or without the presence of a fracture [1]. Fixation with syndesmotic screws is widely considered to be the gold standard for repair, but in recent years, fixation with suture bridge (SB) devices has gained popularity. Lower complication rates, decreased need for implant removal, and faster return to activity have been reported with SB use [2-4]. Suture endobutton constructs allow for the maintenance of physiologic motion at the syndesmosis while screw constructs are rigid [5]. This flexibility allows the SB to remain implanted without the need for a second surgery for removal. Comparisons of the American Orthopedic Foot and Ankle Society (AOFAS), Foot and Ankle Disability Score (FADI), and Olerud-Molander Ankle Score (OMAS) revealed similar post-operative measurements without evidence of chronic instability in both SB and screw constructs [6].

There are mixed results in the literature regarding complications of SB fixations. A study performed in 2009 demonstrated significant soft tissue complications with the use of SB fixation for ankle syndesmosis diastasis with subsequent need for removal [7]. However, a more recent study demonstrated no complications related to SB devices; these findings were maintained at a mean of 14 months [8]. The clinical and radiologic findings of patients receiving SB fixation compared to screw fixation had no significant differences when tibiofibular clear space, medial clear space, and tibiofibular overlap were compared [9]. A recent study utilizing the Physiotherapy Evidence Database (PEDro) checklist for randomized control trials and the Downs and Black checklist for cohort studies determined that grade A evidence exists in support of the use of SF fixation for these injuries [10]. Evaluation of alignment with SB fixation has also been performed. Weber C ankle fractures repaired with SB systems have been shown to achieve a return of the syndesmosis to a normal radiographic pre-injury alignment without evidence of re-displacement in follow-up imaging [11]. The rate of malreduction may increase over time with the use of screw fixation, but the use of SB fixation has been shown to maintain reduction when evaluated with computerized tomography (CT) at least two years after surgery [12].

When evaluating the appropriate method of syndesmotic fixation, several factors should be taken into consideration. A contraindication to SB fixation is known to be osteoporotic bone, as this is widely believed to increase the chances of implant failure. However, an osteoporosis evaluation is not necessarily required after an ankle fracture as it does not appear that osteoporosis is a significant risk factor for ankle fractures [13]. There are limited studies exploring the strength of suture buttons in osteoporotic bone in the ankle; a literature search returned only one study that showed osteoporosis was a factor in the development of infection within one year after SB fixation [14]. While the direct comparison of SB and syndesmotic screws has been widely evaluated, few studies have compared SB construct outcomes among patients of different age groups. This study aims to compare the outcomes of SB fixation among patients ≥ 60 and <60 years of age to determine if the use of SB fixation has greater complication or revision rates in an older cohort compared to a younger one. 

## Materials and methods

Study population 

This was a retrospective review of a consecutive series of 140 patients with ankle fractures who received SB fixation at a single institution, Anne Arundel Medical Center, Annapolis, Maryland, United States, between July 13, 2010, and February 2, 2022. Patient data were obtained from patient records in the EPIC electronic health record (EHR) (Epic Systems Corporation, Verona, Wisconsin, United States). Osteoporosis and osteopenia were defined as patients with a formal diagnosis of these conditions present in the EHR. DEXA (dual x-ray absorptiometry) scores were not performed on each patient. The cutoff of age 60 was chosen based on the clinician's judgment of increased risk of osteoporosis and osteopenia, given that the current guidelines recommend evaluation of all men 70 years or older or postmenopausal women aged 50 or older [15,16].

Surgical technique 

All surgeries were performed by an attending surgeon using a standard medial, lateral, posterior, or combination approach to the ankle as required for appropriate fixation of fracture fragments. No specific institutional protocol for the use of SB fixation was used. Surgeons selected fixation type based on clinical judgment. The suture buttons used were all Arthrex TightRope® (Arthrex, Inc., Naples, Florida, United States). Post-operative care followed standard institutional protocol. 

Study variables 

Age, sex, race, body mass index (BMI), and prevalence of the following comorbidities were examined: osteoporosis, osteopenia, smoking status, diabetes, and renal disease. The affected side, mechanism of injury, Weber classification, type of fracture (proximal fibular, lateral malleolar, bimalleolar, or trimalleolar), primary procedure performed, and other secondary procedures performed were also examined. 

Study outcomes 

Post-operative outcomes of interest included complications, complication type, post-operative neuropraxia, loss of fixation, and loss of fixation requiring revision. Complications of interest included delayed wound healing, infection, hardware, loosening, non-union, and others. 

Complications were grouped based on the provider’s note within the patient’s chart. Delayed wound healing was any instance in which the note described delayed surgical wound healing, soft tissue impingement lesion, or maceration of the skin. Infection was defined as any instance in which the note described cellulitis or a staph infection. Hardware was defined as any instance in which the note described screw exposure. Loosening was defined as any instance in which the note described pin loosening. Non-union was defined as any instance in which the note described a delay in bony consolidation, ossification within syndesmosis, or non-union. Other complications included tendonitis, peptic ulcers, and death. 

Statistical analysis 

Univariate analysis, including chi-square and independent t-tests, was used to assess differences in demographics, injury mechanisms, surgical details, and complication differences between those aged under 60 years and those aged 60 years and older who received SB fixation. Subgroup analysis was then conducted to assess differences in complication rate by age group. Age groups were defined as: < 30 years old, 30-39 years old, 40-49 years old, 50-59 years old, and 60+ years old. A priori power analysis was conducted to determine that this study had adequate sample sizes to detect medium and large effect sizes for continuous and categorical endpoints respectively with 80% power. Cohen’s d was defined as the difference between two means divided by the standard deviation of the data. Cohen described a “d” of 0.20 to be a small effect size, 0.50 to be a medium effect size, and 0.80 to be a large effect size for continuous endpoints [17]. Cohen’s w was defined as a measure of effect size used for chi-squared tests. Cohen described a “w” of 0.10 to be a small effect size, 0.30 to be a medium effect size, and 0.50 to be a large effect size for categorical endpoints [17]. The sample size necessary to detect large effect sizes were 52 and 64 for continuous and categorical endpoints respectively, 128 and 176 for medium effect sizes, and 788 and 1,570 for small effect sizes (Table 1).

**Table 1:** Power Analysis: Sample Size Required to Detect Different Effect Size Represents total N; Cohen’s d: measure of effect size for t-tests; Cohen’s w: measure of effect size for chi-squared tests

Power Analysis	Small (d=0.2)	Medium (d=0.5)	Large (n=0.8)
Continuous Endpoint	788	128	52
Power Analysis	Small (w=0.1)	Medium (w=0.3)	Large (w=0.5)
Categorical Endpoint	1570	176	64

All statistical analyses were performed using R Studio Version 1.4.1717 (Released 2021; RStudio, PBC, Boston, Massachusetts, United States). Statistical significance was assessed at p<0.05. 

## Results

Of the 140 patients with ankle fractures, 104 (74%) patients were younger than 60 years, and 36 (26%) were aged 60 years or older. Demographically, there were no significant differences in sex, race, or BMI between those who were younger than 60 years and those who were 60 or older. However, those who were 60 or older were more likely to have osteoporosis (11.0% vs. 0%; p=0.004). Additionally, there were no significant differences in osteopenia, smoking status, diabetes, or renal disease between those who were younger than 60 years and those who were 60 or older (Table 2).

**Table 2:** Patient Demographics P values <0.05 in bold; Data are expressed as mean ± SD or n (%); BMI, body mass index

Patient Demographics	Younger than 60 (n=104)	60 or Over (n= 36)	P value
Age	41.56 ± 11.53	68.03 ± 7.94	<0.001
Sex			0.420
Female	53 (51.0)	22 (61.1)	
Male	50 (48.0)	14 (38.9)	
Non-White Race	26 (25.0)	6 (16.7)	0.533
BMI (kg/m^2^)	33.30 ± 7.85	32.01 ± 7.83	0.401
Osteoporosis	0 (0)	4 (11.1)	0.004
Osteopenia	2 (1.9)	2 (5.6)	0.591
Smoking Status			0.175
Current	17 (16.3)	2 (5.6)	
Former	29 (27.9)	10 (27.8)	
Never	67 (64.4)	23 (63.9)	
Diabetes	14 (13.5)	9 (25.0)	0.185
Renal Disease	0 (0)	1 (2.8)	0.581

Differences in injury and surgical details were assessed between those who were younger than 60 years and those who were 60 or older. There were no differences in the mechanism of injury, affected side, Weber classification, and primary or other procedures performed between the two groups. However, those who were aged 60 or older sustained significantly more trimalleolar fractures than the younger age group (47.2% vs. 26.0%, respectively; p<0.001) (Table 3).

**Table 3:** Injury and Surgical Details P values <0.05 in bold; Data are expressed as n (%)

Injury and Surgical Details	Younger than 60 (n=104)	60 or Over (n=36)	P value
Affected Side			0.605
Right	58 (55.8)	18 (50.0)	
Left	44 (42.3)	18 (50.0)	
Mechanism of Injury			0.164
Slip/Fall	78 (75.0)	28 (77.8)	
Sports	16 (15.4)	2 (5.6)	
Other	9 (8.7)	6 (16.7)	
Weber Classification			0.441
B	39 (37.5)	13 (36.1)	
C	37 (35.6)	17 (47.2)	
C (Maisonneuve)	22 (21.2)	5 (13.9)	
Proximal Fibular	5 (4.8)	3 (8.3)	0.732
Lateral Malleolar	30 (28.8)	7 (19.4)	0.346
Bimalleolar	39 (37.5)	9 (25.0)	0.219
Trimalleolar	27 (26.0)	17 (47.2)	0.037
Primary Procedure			0.485
Ankle Arthroscopy	4 (3.8)	0 (0)	
Fracture Fixation	96 (92.3)	35 (97.2)	
Repair of Syndesmosis	3 (2.9)	1 (2.8)	
Other Procedure	21 (20.2)	6 (16.7)	0.809
Deltoid Ligament Repair	12 (11.5)	5 (13.9)	0.823

Postoperatively, there were no statistically significant differences in complication rates, which occurred in 14 (13.5%) patients under 60 years and six (16.7%) patients aged 60 or over (p=0.860). Further, no significant differences in rates of postoperative neuropraxia (p=1.000) or other complication types, including loss of fixation, or loss of fixation requiring surgery were observed between groups (p=0.079, Table 4).

**Table 4:** Complications P values <0.05 in bold; Data are expressed as n (%)

Complication Details	Younger than 60 (n=104)	60 or Over (n=36)	P value
Complication	14 (13.5)	6 (16.7)	0.860
Complication Type			0.079
Delayed Wound Healing	7 (6.7)	1 (2.8)	
Infection	1 (1.0)	1 (2.8)	
Hardware	0 (0)	1 (2.8)	
Loosening	1 (1.0)	0 (0)	
Non-Union	4 (3.8)	0 (0)	
Other	1 (1.0)	3 (8.3)	
Postoperative Neuropraxia	3 (2.9)	1 (2.8)	1
Loss of Fixation	3 (2.9)	2 (5.6)	0.808
Loss of Fixation Requiring Revision	2 (1.9)	2 (5.6)	0.531

**Figure 1:**
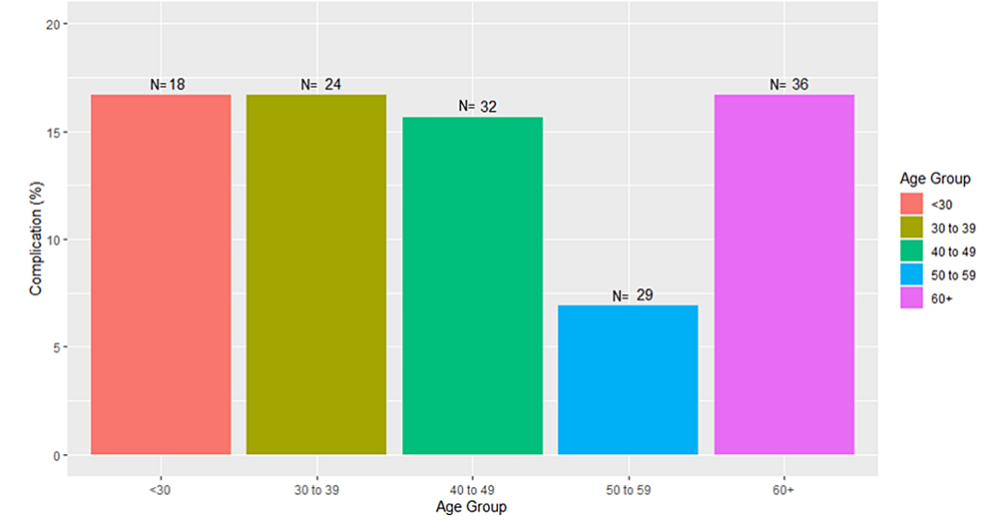
Complication Rate by Age Group

Finally, subgroup analysis assessed the rate of complications for the following age groups: < 30 years old, 30-39 years old, 40-49 years old, 50-59 years old, and 60+ years old. A total of 16.8% of patients younger than 30 years, 16.8% of patients in the age group of 30-39 years, 15.6% of patients in the age group of 40-49 years, and 16.7% of patients that were 60 or older experienced a complication, while only 6.9% of patients in the age group of 50-59 years experienced a complication. Overall, there was no significant difference between the rates of complications across these age groups (Figure 1).

## Discussion

The results of the current study demonstrate that patients aged 60 years and above undergoing SB fixation for the treatment of syndesmotic injuries experience similar complication rates as those under the age of 60. While surgeons may hesitate to use SB fixation in older populations at risk for decreased bone quality, these findings suggest absolute age cutoffs are not warranted in the selection of fixation technique. To our knowledge, this is the first study to specifically compare the outcomes of SB fixation across age groups. 

To date, the majority of studies have compared the outcomes of SB versus screw fixation in the treatment of syndesmotic injuries. A retrospective review of 52 patients aged 65 years or younger receiving either a syndesmotic screw or SB fixation, with SB patients achieving better restoration of fibular rotation and increased tibiofibular space compared to the screw fixation group [18]. Similarly, a randomized control trial of 54 patients aged 18-60 years also comparing SB to screw fixation found that patients receiving knotless SB constructs had an earlier return to sports and good clinical outcomes with the only statistically significant variable being the type of construct used [19]. Further, a randomized control trial of 62 patients, 41 of which were available for 10-year follow-up, established that the long-term outcomes of SB fixation had similar clinical outcome scores to syndesmotic screws without evidence of chronic instability [6]. The mean ages of patients in this study were 44.4 for SB fixation and 47.2 for screw fixation. While the current study did not evaluate long-term clinical outcomes, our results demonstrating similar complication rates across SB fixation age groups suggest that older populations may also safely undergo this fixation technique. Given the demonstrated benefits of SB fixation in younger populations, follow-up studies are warranted to confirm whether older patients undergoing SB fixation experience similar functional improvements as the younger population. 

Although prior studies have not evaluated the outcomes of SB fixation for the treatment of syndesmotic injuries across age groups, this approach has been used to study surgical repair of ankle fractures in general. In a retrospective chart review, 2353 ankle fractures treated by open reduction and internal fixation were evaluated in two groups of patients aged 65-79 years and 80-89 years with no increased risk of complications or 30-day mortality detected in either group when controlling for comorbidities [20]. The results of the current study suggest a similar, non-significant difference in complication rates across age groups exists in patients undergoing SB fixation. Physician preference and judgment should therefore be employed when deciding if a patient is a candidate for SB fixation independent of the patient’s age. 

While osteoporosis was a statistically significant factor between the two groups, this is likely due to the prevalence of osteoporosis in patients over 65 [21]. No patients under 60 in our study had a diagnosis of osteoporosis at the time of surgical repair. However, osteoporosis is not the only risk factor that increases the risk of complications in the older population. A retrospective chart review of 90 patients with or without diabetes aged 18-65 years with ankle fractures treated surgically were compared and it was seen that wound complications were significantly increased in patients with diabetes mellitus and increased BMI [22]. A study utilizing the National Surgical Quality Improvement Program (NSQIP) database evaluated significant predictors on outcomes for patients receiving open reduction and internal fixation (ORIF) for open ankle fractures over a five-year period and determined that concurrent chronic obstructive pulmonary disease (COPD) increased mortality while peripheral vascular disease, American Society of Anesthesiologists (ASA)>3, and open or contaminated wounds significantly increased the likelihood of wound complications [23]. In our results, no other demographics or comorbidities such as gender, race, diabetes mellitus, smoking status, or renal disease were statistically significant between the groups. While there were no significant differences in comorbidities for either group, it is important to evaluate whether surgical fixation will heal appropriately in patients with these comorbid conditions, potentially causing increased complications and requiring repeat surgeries. 

Another potential benefit of SB use in syndesmotic injuries is the decrease in the number of surgical procedures required compared to syndesmotic screws. A systematic review and meta-analysis comparing SB to screw fixation revealed a significantly lower incidence of reoperation, fewer cases of malreduction, and improved AOFAS [24]. The results of the current study build on this finding, showing that no significant differences in reoperation exist between younger and older patients undergoing SB fixation. The need for reoperation in screw fixation may contribute to increased cost of care in those patients, which should be taken into consideration when deciding whether SB or screw fixation is preferable. 

There are limitations to this retrospective chart review performed at a single institution. Taking into consideration the small sample size, this study is limited and may not be representative of all healthcare systems or patient populations. Expansion of this study to other institutions or over a longer period can strengthen the results or reveal statistically significant differences that were not noted here but are more applicable to larger, more varied populations. Further, there are other clinical factors that were not analyzed in the current study that may confound our results. The Lauge-Hansen classification system was not utilized; this may have led to a simplification of fracture patterns and their representation in each age group. Additionally, comorbidities were assessed using International Classification of Diseases, 10th Revision (ICD-10) codes. Therefore, we were unable to assess important details of conditions such as the level of control and associated symptoms present in diabetic patients. Furthermore, in clinical practice, the development of osteoporosis is not routinely monitored in every patient. Therefore, as a retrospective chart review, the true prevalence of osteoporosis in this study population is not defined, limiting the utility of this study with regard to osteoporotic patients. A larger, prospective study that includes the use of DEXA scoring in the protocol may be able to further compare the use of SB fixation in osteoporotic patients and those without that diagnosis. Another limitation is the use of age 60 as the cutoff. While surgeons involved in this study feel that patients over 60 may have increased risk based on warnings associated with the use of suture fixation, other institutions may use another age cutoff, such as 65, leading to differing results than the ones shown here. This may have contributed to the presence of selection bias in our results, as evidenced by the smaller sample of patients aged 60 and above who received SB fixation. Finally, half of the osteoporotic patients in this study sustained a trimalleolar fracture, which was a larger proportion than in the non-osteoporotic group. This may be due to the small size of the osteoporotic group, which only contained four patients. Given the small number of verified osteoporotic patients, this study is not sufficiently powered to determine why this population sustained more trimalleolar fractures than the younger cohort. A larger, multi-center prospective study with a well-defined osteoporotic population is needed to further explore this finding. 

## Conclusions

The rates and types of complications among patients younger than 60 years and patients aged 60 years and above are not significantly different between surgical fixation of distal ankle fractures and SB construct. SB fixation may be a suitable method of treatment regardless of patient age. Surgeons must continue to evaluate whether SB fixation is appropriate on a patient-specific basis, but absolute age cutoffs do not appear warranted in the selection of fixation technique. 
